# Epithelial Electrolyte Transport Physiology and the Gasotransmitter Hydrogen Sulfide

**DOI:** 10.1155/2016/4723416

**Published:** 2016-01-20

**Authors:** Ervice Pouokam, Mike Althaus

**Affiliations:** ^1^Institute for Veterinary Physiology and Biochemistry, Justus-Liebig University, Frankfurter Strasse 100, 35392 Giessen, Germany; ^2^Institute for Animal Physiology, Justus-Liebig University, Heinrich-Buff-Ring 26, 35392 Giessen, Germany

## Abstract

Hydrogen sulfide (H_2_S) is a well-known environmental chemical threat with an unpleasant smell of rotten eggs. Aside from the established toxic effects of high-dose H_2_S, research over the past decade revealed that cells endogenously produce small amounts of H_2_S with physiological functions. H_2_S has therefore been classified as a “gasotransmitter.” A major challenge for cells and tissues is the maintenance of low physiological concentrations of H_2_S in order to prevent potential toxicity. Epithelia of the respiratory and gastrointestinal tract are especially faced with this problem, since these barriers are predominantly exposed to exogenous H_2_S from environmental sources or sulfur-metabolising microbiota. In this paper, we review the cellular mechanisms by which epithelial cells maintain physiological, endogenous H_2_S concentrations. Furthermore, we suggest a concept by which epithelia use their electrolyte and liquid transport machinery as defence mechanisms in order to eliminate exogenous sources for potentially harmful H_2_S concentrations.

## 1. Introduction

*“All things are poisons, for there is nothing without poisonous qualities. It is only the dose which makes a thing poison.”* This famous quote from Paracelsus (1493–1541) is still important for research in physiology and especially for recent investigations on a toxic gas, hydrogen sulfide, and its potential role as a physiologically relevant signalling molecule.

Hydrogen sulfide (H_2_S) is well known as an environmental chemical threat and even more for its unpleasant smell of rotten eggs. The odour threshold for H_2_S is about 0.003–0.02 ppm and concentrations above 50 ppm have toxic effects such as irritations of the eye and respiratory tract [1]. At 150–200 ppm H_2_S, the olfactory sense for this gas is lost and higher concentrations lead to the formation of pulmonary oedema, unconsciousness, and eventually death [1]. The toxic effects of H_2_S are mainly based on the inhibition of the mitochondrial respiratory chain, especially cytochrome c oxidase [2, 3]. However, consistent with the principle of Paracelsus, research of the past decade has revealed that cells endogenously produce small amounts of H_2_S which are not simply a metabolic by-product and play an important role in cellular signalling processes [4]. Similar to nitric oxide (NO) or carbon monoxide (CO), H_2_S has therefore been classified as a “gasotransmitter,” a gaseous cellular signalling molecule [4, 5]. Furthermore, a therapeutic potential for low-dose H_2_S has been discovered [4] and H_2_S-releasing pharmacological compounds have been designed [6] and are currently evaluated as potential therapeutics in various models of disease [7].

A major challenge for cells and tissues is the maintenance of physiological (low) concentrations of H_2_S in order to prevent potential toxicity. In this review article, we describe epithelial reactions to H_2_S. We focus on epithelia of the respiratory and intestinal tract since these tissues are predominantly exposed to a variety of exogenous and potentially dangerous sources for H_2_S, that is, inhaled H_2_S in the lung and microbiota-generated H_2_S in the gut. Furthermore, epithelial cells endogenously produce low concentrations of H_2_S with potential implications for cellular signalling processes. In line with the principle of Paracelsus, epithelia therefore have to find a balance between potentially toxic exogenous and physiological, endogenous H_2_S concentrations. In the following sections we will describe the chemistry as well as sources of H_2_S to which epithelia are exposed, their reactions to exogenous and endogenous H_2_S, and potential physiological/pathophysiological implications with respect to epithelial function.

## 2. Hydrogen Sulfide: Properties, Exogenous Sources, and Enzymatic Production

### 2.1. Chemical Properties of Hydrogen Sulfide

H_2_S is a colourless and flammable gas characterized by its rotten eggs or blocked sewer smell. At 20°C, one gram of H_2_S will dissolve in 242 mL water. Temperature and time influence the concentration of H_2_S; temperature elevation increases the solubility of this gas. Oxidation occurring over time in solution leads to precipitation of elemental sulfur, giving a cloudy aspect to the solution (for review see [4]). Experimental work with this molecule is complicated since H_2_S evaporates easily from aqueous solutions with a half-life on the minute time-scale [4, 8, 9]. In solution, H_2_S is a weak acid, dissociating into the hydrosulfide anion or thiolate form HS^−^ and the sulfide anion S^2−^ building the following equilibrium:

(1) HS2⟷HS−⟷S2−

with p*K*a1 and p*K*a2 values of 6.9 and >12, respectively.

Consequently, at 37°C and physiological pH, there are almost equal amounts of H_2_S and HS^−^ (but no S^2−^) in cells and tissues and nearly a 20% H_2_S/80% HS^−^ ratio in extracellular fluid or plasma [4]. H_2_S is highly lipophilic with a dipole moment of 0.97 [10], allowing for a rapid cell membrane permeation [11] and hence potential interference with cell respiration. Diffusion of H_2_S into the cells is hence a primary problem of respiratory and intestinal epithelia which are predominantly exposed to exogenous and potentially dangerous levels of H_2_S.

### 2.2. Exogenous and Endogenous H_2_S Sources

#### 2.2.1. Exogenous H_2_S Sources

**Figure 1:**
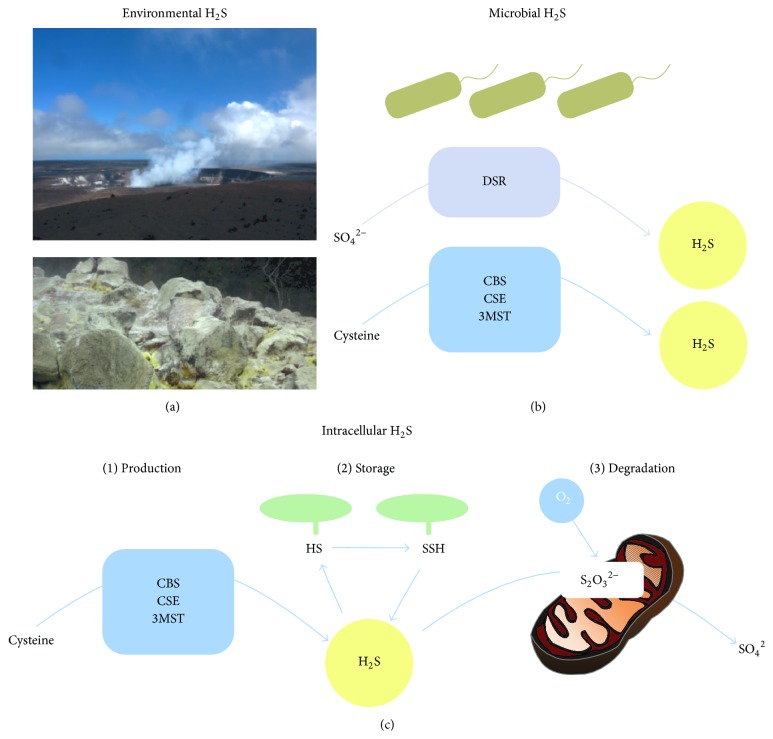
Sources of hydrogen sulfide. (a) Environmental hydrogen sulfide (H_2_S) occurs due to geothermal activities such as volcanoes, sulfur deposits, or sulfur springs. The pictures show the Halema‘uma‘u crater (top) and sulfur deposit (bottom) on Hawaii Big Island. (b) Microbial production of H_2_S. Sulfate-reducing bacteria dissimilate sulfate into H_2_S via dissimilatory sulfite reductase (DSR). Mesophilic bacteria use orthologues of the mammalian H_2_S-generating enzymes cystathionine-*β*-synthase (CBS), cystathionine-*γ*-lyase (CSE), and 3-mercaptopyruvate sulfurtransferase (3MST) in order to generate H_2_S from cysteine. (c) Mammalian cells produce H_2_S from cysteine via CBS, CSE, and 3MST (1). H_2_S can be stored in a reductant-labile intracellular pool as bound sulfane sulfur (2). The degradation of H_2_S occurs via oxidative metabolic pathways in mitochondria (3).

There are organic and inorganic sources of H_2_S (Figure 1). The pulmonary epithelium can be exposed to H_2_S by inhalation of environmental H_2_S gas (Figure 1(a)). H_2_S may be released by volcanoes as natural gas or contained in sulfur deposits or (healing) sulfur springs. Additional significant H_2_S sources are industrial processes such as petroleum refinery, rayon manufacturing, and paper, swine, and pulp mill industry [4]. 125,000 employees in 73 industries of the United States are potentially exposed to H_2_S [4].

In the intestinal tract, methionine- or cysteine-rich diet may end up as H_2_S production/release. The intestinal flora may produce H_2_S via sulfate-reducing bacteria (SRB) (Figure 1(b), Table 1). SRBs use several substrates like short-chain fatty acids, other organic acids, or alcohol as electron donors for reduction of sulfate or other oxidized sulfur molecules to produce H_2_S. Furthermore, endogenous substrates in the large intestine, such as sulfated polysaccharides (sulfomucins), can be used by the microflora for H_2_S generation [12, 13]. Sulfates may also be directly delivered by food such as dried fruits, nuts, bread, wine, or brassica vegetables. The key bacterial enzyme used during these processes is dissimilatory sulfite reductase [14]. Furthermore, Shatalin and colleagues demonstrated that clinically relevant and pathogenic nonsulfur bacteria such as* Bacillus anthracis*,* Pseudomonas aeruginosa*,* Staphylococcus aureus,* and* Escherichia coli* produce H_2_S endogenously [15]. These species contain orthologues of the mammalian H_2_S-generating enzymes cystathionine-*β*-synthase (CBS), cystathionine-*γ*-lyase (CSE), and 3-mercaptopyruvate sulfurtransferase (3MST) and metabolize cysteine [15]. Overall, up to millimolar H_2_S concentrations can occur in the content of human colon [13, 16] due to food compounds and activity of the intestinal flora.

In experimental studies, exogenous H_2_S is mainly administered by “H_2_S donors.” Sulfur salts such as NaHS, Na_2_S, and Lawesson's reagent are routinely used for H_2_S delivery. However, these molecules are fast donors (in fact, these salts readily dissociate), contrasting with a continuous and more physiologically relevant production and release of H_2_S which may occur in the body. Synthetic slow-releasing H_2_S donors are, for example, GYY4137 or ATB-429 (for review, see [6, 17]). Natural H_2_S donors are, for example, compounds in garlic extracts such as diallyl poly(di,tri)sulfides which slowly release H_2_S. Also the sulfur-compounds S-allylcysteine (SAC) and allicin are found in garlic. These organic polysulfides may react with intracellular thiols like glutathione (GSH) to produce H_2_S [4].

#### 2.2.2. Endogenous, Intracellular H_2_S

H_2_S is endogenously produced by various cell types, including epithelial cells, as a result of L-cysteine metabolism. For the detailed biochemistry of cellular H_2_S production pathways, the reader is referred to excellent recent review articles [18, 19]. In the past years, a growing body of evidence suggests a concept by which intracellular H_2_S concentrations are determined by (1) its enzymatic production; (2) its intracellular storage as bound sulfane sulfur; and (3) its oxidative degradation by mitochondria.

*(1) Enzymatic H*_*2*_ *S Production*. H_2_S is generated by three enzymes: CBS, CSE and 3MST. L-cysteine is the substrate for pyridoxal-5′-phosphate (vitamin B6) dependent CBS and CSE. However, it can be synthesized from L-methionine through the transsulfuration pathway catalysed by methionine adenosyltransferase (MAT) and glycine N-methyltransferase (GNMT). Serine is then transferred to homocysteine leading to cystathionine, a reaction catalysed by CBS. CSE converts cystathionine to cysteine. Cysteine may be converted to L-cystine which in turn will be converted to thiocysteine and further to H_2_S. CSE and CBS together catalyse the production of H_2_S from cysteine. The abovementioned processes occur within the cytosol. Cysteine aminotransferase (CAT) and 3-mercaptopyruvate sulfur transferase (MPST), both found in cytosol and mitochondria, catalyse the conversion of cysteine to 3-mercaptopyruvate and H_2_S, respectively [4, 9, 20].

In addition, there is enzyme-free generation of H_2_S; however, this represents only a minor source of this gasotransmitter. Here, oxidation of sulfide produces thiosulfate, which in turn interacts with intracellular thiols such as GSH to release H_2_S. 

*(2) Intracellular H*_*2*_ *S-Storage*. Enzymatically produced H_2_S can be intracellularly stored as bound sulfane sulfur [18]. This occurs by the oxidative formation of hydrodisulfides or persulfides, for example, by modification of the sulfur of cysteine residues in proteins, which establish a reductant-labile pool of sulfane sulfur [18, 21]. A study by Shibuya and colleagues demonstrated that HEK293-F cells expressing 3MST contained larger amounts of sulfane sulfur than nontransfected cells or cells expressing enzymatically inactive 3MST [22]. This indicates that enzymatically produced H_2_S is transformed into bound sulfane sulfur, thereby reducing the concentration of free intracellular H_2_S. 

*(3) Mitochondrial H*_*2*_ *S-Degradation*. H_2_S affects mitochondrial respiration by inhibiting cytochrome c oxidase [2] with an IC_50_ value of 0.32 *μ*M in colonic epithelial cell homogenates [27]. Despite this finding, colonic epithelial cells are surprisingly resistant to even millimolar H_2_S concentrations [13, 27] due to a detoxifying sulfide metabolism [13]. Remarkably, low (micromolar) concentrations of H_2_S are even able to stimulate cellular respiration and represent an energy substrate for epithelial cells (for excellent review see [13]). H_2_S is metabolized in mitochondria by the sulfide oxidation pathway [18, 28]. In the presence of oxygen, sulfide:quinone oxidoreductases (SQR) oxidize H_2_S to persulfide which is subsequently oxidized into sulfite via dioxygenase. Sulfite is further transferred into thiosulfate by rhodanese. Thiosulfate reductase and sulfite oxidase eventually metabolize thiosulfate into sulfate which is excreted. Interestingly, epithelial tissues which are predominantly exposed to H_2_S, such as colon epithelia, have a particular high metabolic, H_2_S-detoxifying capacity [29, 30].

The oxidative metabolism of H_2_S in mitochondria implies that concentrations of free H_2_S in normoxic tissues are low. The group of Olson demonstrated with H_2_S-specific amperometric sensors that endogenous H_2_S production inversely correlates with oxygen concentrations [28, 31–33]. Hence, significant amounts of H_2_S are only produced in anoxic environments and rapidly disappear in the presence of oxygen. This dynamic relationship between oxygen and H_2_S indicates the necessity to maintain free H_2_S concentrations low and, furthermore, led to the hypothesis that H_2_S might be a cellular oxygen sensor [28, 34, 35].

In sum, recent evidence suggests that there is a dynamic intracellular life-cycle of H_2_S. Its enzymatic production is antagonized by storage as bound sulfane sulfur and oxidative metabolism, thereby limiting the concentration of free H_2_S to the submicromolar range [36, 37]. Aside from the intracellular biochemical mechanisms to maintain low H_2_S concentrations, there are epithelial mechanisms which prevent the exposure to potentially dangerous, high H_2_S concentrations. This will be described in the next sections.

## 3. Epithelial Reactions to H_2_S

Similar to nervous, connective and muscle tissues, epithelia are a basic type of animal tissue. They are continuous sheets of tightly packed cells lining the surfaces and cavities of the body [38]. Epithelial cells are attached to each other by protein complexes such as tight junctions. The structure of an epithelium depends on the morphology of its cells and on the amount of cell layers it is made of. In monolayer epithelia (also called simple epithelia) the cells rest on the basal membrane. Flat and scale-like cells are characteristic of simple squamous epithelia. Cube-shaped cells constitute the simple cuboidal epithelium while simple columnar epithelia (gastrointestinal tract) are built of column-shaped cells. The cell nuclei may be disposed at different levels allowing for a pseudocellular stratification, a characteristic of the pseudostratified columnar epithelium which can be ciliated (trachea, upper respiratory tract) or not (penile urethra). When made of many layers, the epithelium is qualified as “stratified.” Here, the shape of the upper most cells determines the type of epithelium. Hence, stratified squamous keratinized (skin) or not (oesophagus), stratified cuboidal (ducts of sweat glands), or stratified columnar (conjunctiva) epithelia can be distinguished. The lower most cell layers may comprise different types of cells. A further characteristic of stratified epithelia, besides keratinization, is their ability to contract and stretch. For the latter, they are called transitional epithelia (urothelium).

Epithelia are multifunctional structures which protect from mechanical forces or physical trauma, from desiccation or toxins. They act as barriers against pathogens, regulating exchanges of, for example, water and electrolytes between the* milieu intérieur* and the* milieu extérieur*. The epithelia of the lung or the digestive tract are especially faced with building a protective barrier towards the outer environment on the one hand and at the same time allowing for efficient exchange, such as the respiratory gases or food components, on the other. Vectorial transport of electrolytes and water is a fundamental feature of these epithelia, regulating the diffusion barrier for respiratory gases in the lung, or exchanging nutrients and water from intestinal content. Furthermore, these epithelia use secretory mechanisms as a defence reaction against potentially harmful substances by flushing their outer surfaces. Hence, the epithelial electrolyte and water transport machinery represents both a key feature for normal physiology of these organs and primary defence mechanism. In the following section we discuss the impact of H_2_S, in both potentially harmful and physiological concentrations, on electrolyte and water transport mechanisms in intestinal and pulmonary epithelia.

### 3.1. H_2_S and Intestinal Epithelia

#### 3.1.1. Intestinal Epithelial Responses to Exogenous H_2_S

The intestinal epithelium fulfils the above mentioned epithelial functions, with a more or less secretory or absorptive profile depending on the segment. The determination of intestinal epithelial functions finds its origins in its cellular organization. Intestinal epithelia are organized into finger-like protrusions called villi, absent in the colon, and invaginations called crypts of Lieberkühn (short: crypts). Four types of cells constitute the intestinal epithelium: enterocytes, goblet cells, Paneth cells, and enteroendocrine cells. The Paneth cells, absent in the colon, are located in the crypts and promote defence by their antimicrobial secretions. Also, pluripotent stem cells are located in the crypts; their mitosis generates the other cells, which migrate up the crypt-villus axis and differentiate. The goblet cells produce and secrete mucus, protecting against shear stress and chemical damage [38]. Enteroendocrine cells produce hormones or peptides for digestion or chemical sensors for digestive reflexes. Enterocytes are polarized cells representing the absorptive cell lineage. They carry an apical brush border which is responsible for enzymatic digestion, as well as ion, water, and nutrient uptake. These transport pathways may be transcellular passive (facilitated or not), active, or paracellular through cell junctions such as tight junctions.

Intestinal ion transport, which can be switched from absorption into secretion of water and electrolytes, is controlled by neurotransmitters and hormones, but also gasotransmitters such as CO, NO, H_2_S, and nitroxyl (HNO) [39, 40].

**Figure 2:**
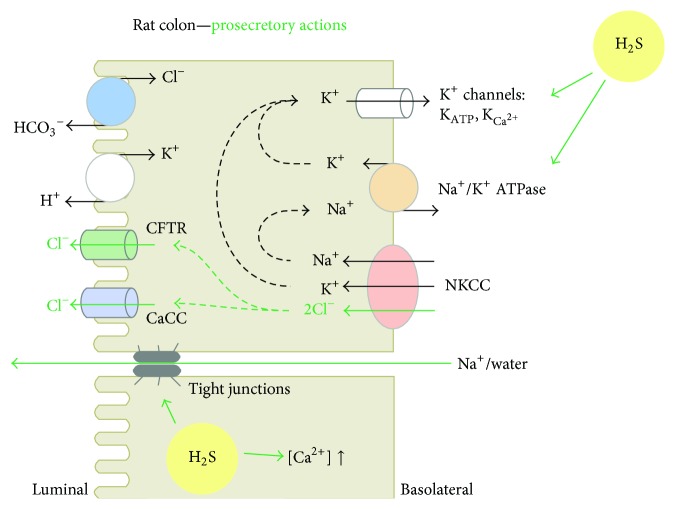
Epithelial ion transport responses to exogenous hydrogen sulfide in rat colon. Channels and transport mechanisms activated by H_2_S in the rat colonic epithelium. Anion (Cl^−^) secretion through the cystic fibrosis transmembrane conductance regulator (CFTR) or calcium-dependent chloride channels (CaCC) is enabled after building up a cytosolic anion potential, which results from the activation of basolateral transporters notably: the Na^+^/K^+^-ATPase maintaining the K^+^ concentration gradient between the intracellular and the extracellular spaces; the Ca^2+^-dependent (K_Ca^2+^_) and ATP-sensitive (K_ATP_) K^+^ channels, responsible for the driving force allowing for uptake of Cl^−^ via the basolateral Na^+^-K^+^-Cl^−^ cotransporter type 1 (NKCC1). H_2_S donors induce activation of the Na^+^/K^+^-ATPase and basolateral K^+^ conductance, enabling intracellular accumulation of Cl^−^, which will be secreted via the CFTR or CaCC. Also, the apical Na^+^/Ca^2+^ exchanger was activated in parallel with increase in paracellular permeability.

The colonic epithelium absorbs water and electrolytes under basal conditions; however, distention of the gut wall by intestinal content induces electrolyte secretion in order to generate a fluid film which facilitates the protrusion of the content. The switch to secretion is also observed after exposure of the epithelium to bacterial products and observed in the development of secretory diarrhoea under pathophysiological conditions. Secretion by the colonic epithelium is predominantly driven by a transepithelial secretion of chloride ions into the lumen, which osmotically facilitates liquid secretion. In order to secrete chloride across the apical membrane of the epithelial cells, the basolateral membrane has to establish a driving force for anion efflux via anion channels (e.g., cystic fibrosis transmembrane conductance regulator, CFTR, or Ca^2+^-sensitive chloride channels) located in the apical membrane (Figure 2). In the basolateral membrane, potassium channels generate the driving force for chloride secretion by maintaining the negative membrane potential which is dominated by a potassium diffusion potential [41, 42]. The Na^+^/K^+^-ATPase maintains the potassium concentration gradient between the intra- and the extracellular space as a prerequisite for the establishment of the potassium diffusion potential (Figure 2).

H_2_S affects key ion transport processes within the colonic epithelium. In rat, guinea pig, and human colon preparations H_2_S (applied by sulfur salts) elicits a secretory response [43–45]. In rat colon, H_2_S activates the apical membrane Na^+^/Ca^2+^ exchanger extruding Ca^2+^ (Figure 2). It also induces an increase in cytosolic Ca^2+^ concentration as a result of the release of stored Ca^2+^ from intracellular organelles via IP_3_-receptors or ryanodine receptors (RyR). Cytosolic accumulation of Ca^2+^ triggers the opening of basolateral Ca^2+^-dependent potassium channels (K_Ca_). Furthermore, an ATP-sensitive basolateral potassium (K_ATP_) conductance is activated in the presence of H_2_S. The K_ATP_ channels carrying this conductance may consist of the following combinations: Kir6.1/SUR1, Kir6.1/SUR2B, Kir6.2/SUR1, or Kir6.2/SUR2B [46]. They also exert a protective function against energy depletion as their inhibition by glibenclamide under this condition causes a huge increase in the colonic epithelium conductance [46]. The protective characteristic of K_ATP_ channels was also demonstrated in rats with colitis as their blockade with glibenclamide worsened the disease and increased mortality [47].

Both basolateral potassium conductance (K_ATP_ and K_Ca_) build the driving force for apical chloride efflux (Figure 2). Furthermore, the Na^+^/K^+^-ATPase is also activated by H_2_S [45]. In addition to the secretion of chloride, potassium is also secreted and the epithelial permeability increases [43, 48]. As a consequence of these secretion processes, sodium ions move paracellularly to the lumen (for electroneutrality) and water follows osmotically.

**Figure 3:**
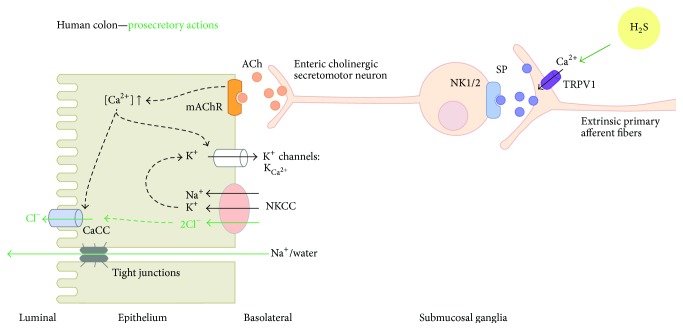
Exogenous hydrogen sulfide stimulates electrolyte secretion in human colon via activation of submucosal neurons. H_2_S stimulates TRPV1 channels in extrinsic primary afferent fibres, which leads to the release of substance P (SP). SP binds to neurokinin receptors (NK) 1 and 2 of enteric cholinergic secretomotor neurons in submucosal ganglia. The subsequently released acetyl choline (ACh) binds to muscarinergic acetyl choline receptors (mAChR) in the epithelial cells. This stimulates a rise in intracellular calcium concentrations which triggers electrolyte secretion by activating calcium-dependent chloride (CaCC) and potassium (K_Ca^2+^_) channels.

Aside from the direct effect of H_2_S on ion channels and transporters within the epithelium, exogenous H_2_S indirectly stimulates chloride secretion by activating secretomotor neurons in human and guinea pig colon mucosa/submucosa preparations [44] (Figure 3). This is supported by the facts that (1) the H_2_S-induced secretory response in these preparations is inhibited by tetrodotoxin and (2) it is absent in the human colon epithelial cell line T84 [44]. The reason for the different actions of H_2_S in rat and human/guinea pig colon preparations is unknown but might be explained by differences in the species. In human/guinea pig colon, H_2_S activates the transient receptor potential vanilloid receptor 1 (TRPV1) in extrinsic primary afferent fibres, which results in a release of substance P [49]. Substance P binds to neurokinin receptors (NK) in enteric cholinergic secretomotor neurons [49]. The subsequent release of acetylcholine by these neurons stimulates epithelial chloride secretion via a muscarinergic receptor- and calcium-mediated signalling axis (Figure 3).

In addition to the described effects of H_2_S on the secretion of chloride ions, H_2_S activates bicarbonate secretion which in turn neutralizes excess acid produced in the gastrointestinal lumen thereby protecting from gastric or duodenal mucosa ulcers [50, 51]. Furthermore, H_2_S increases mucus production from goblet cells, reversing inflammation-associated mucus deficiency [52].

The secretory response to exogenous H_2_S could be interpreted as a defence mechanism of the intestinal epithelium which prevents potentially harmful H_2_S concentrations which might exceed the oxidative capacities of the epithelial cells. Since the oxidative capacity is particularly high in intestinal epithelia [30] the secretory response would be triggered by rather high exogenous H_2_S concentrations. In sum, exogenous H_2_S triggers a secretory epithelial response in the intestine which prevents toxic effects of H_2_S as well as onset of inflammatory processes by flushing the exogenous sources for H_2_S from the epithelial wall.

#### 3.1.2. Endogenous H_2_S Production in Intestinal Epithelia

Gut tissue produces and releases H_2_S [53] and the H_2_S-generating enzymes CBS and CSE have been found within rat colonic epithelium [43]. The local H_2_S concentration within the intestinal wall is unknown but the production rate of H_2_S in rat ileum is in the range of 12 nmol/min/g tissue [54]. Consistent with the described prosecretory actions of H_2_S, inhibitors of CBS and CSE dose-dependently decreased basal anion secretion across rat distal colon preparations, indicating that endogenous H_2_S production might contribute to basal (secretory) ion transport mechanisms in the epithelium [43]. These observations seem to contrast the principle of oxidative degradation of H_2_S under normal physiological conditions (see Section 2.2.2); however, the environment of the gut might generate local, even subcellular anoxic milieus which would allow for enzymatic H_2_S production. Whether or not endogenous H_2_S influences basal secretory processes via the same mechanisms and ion channels/transporters as exogenous H_2_S is unknown. Furthermore, the administration of the H_2_S “precursor” L-cysteine stimulated a secretory response in guinea pig colon preparations, which was sensitive to CBS and CSE inhibitors as well as the TRP channel inhibitor capsaicin [44]. This indicates that the indirect secretory epithelial response in this preparation (see Section 3.1.1) does occur not only due to exogenous H_2_S, but also as a result of enhanced intracellular H_2_S production in submucosal neurons. Whether such endogenous H_2_S production in submucosal neurons occurs under physiological (normoxic) conditions and contributes to ion transport regulation is unknown.

#### 3.1.3. H_2_S in Intestinal Epithelia: Pharmacological Aspects

The local concentration of H_2_S as well as the velocity of the H_2_S-generating pathway determines whether H_2_S acts as a prosecretory (as described above) or as an antisecretory agent. This was demonstrated by assessing chloride secretion by rat enterocytes using different H_2_S-generating compounds: GYY4137 as a very slow releaser, L-cysteine and diallyl trisulfide as relatively slow releasers, and NaHS as a fast releaser. The last two compounds activated chloride secretion whereas L-cysteine and GYY4137 induced the opposite (antisecretory) effect [48]. Such concentration-dependent proabsorptive/prosecretory epithelial actions have already been described for NO and indicate that the kinetics of production, degradation, and even formation of reactive intermediates determines the physiological effects of gasotransmitters. These facts should be considered when evaluating (1) the contribution of (endogenous and exogenous) H_2_S to the pathogenesis of diseases as well as (2) H_2_S-releasing molecules for therapeutic purposes.

For example, pro- and anti-inflammatory effects are ascribed to H_2_S (for review, see [4]). In rat models of colitis, inflammation was resolved by NaHS, Lawesson's reagent, and diallyl trisulfide. These H_2_S donors achieved such a positive action by downregulating the expression of proinflammatory cytokines (IL-1*β*, TNF, INF*γ*, IL-12, and IL-13) and of chemokines (for review, see [7]). This is consistent with the observed therapeutic effects of, for example, the slow-release ATB-429 in a mouse model of colitis [55]. By contrast, other studies describe detrimental effects such as proinflammatory actions or impairment of gastrointestinal integrity [56–58].

In a rat model of colitis, the secretory response to H_2_S was reduced in colon epithelial preparations. This reduction was prevented when the animals were fed on a S-reduced diet (which reduces microflora-mediated H_2_S formation), but not by inhibitors of H_2_S-generating enzymes [59]. This is consistent with an enhanced number of SRBs and faecal H_2_S production in patients with ulcerative colitis [60, 61]. These findings indicate that alteration in exogenous, that is, colonic, microflora-derived H_2_S, determines the efficacy of H_2_S-liberating molecules in diseased tissues. This appears to be reasoned in a desensitisation of the intestinal epithelium to repeated exposure to H_2_S [43]. Again, this example demonstrates that the kinetics of H_2_S production and local concentration are important determinants of epithelial reactions to this molecule.

### 3.2. H_2_S and Pulmonary Epithelia

#### 3.2.1. Pulmonary Epithelial Responses to Exogenous H_2_S

**Figure 4:**
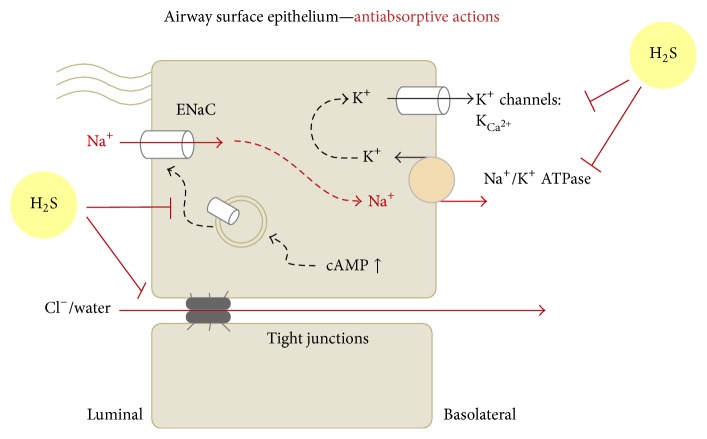
Airway epithelial ion transport responses to exogenous hydrogen sulfide. In airway surface epithelia, H_2_S decreases basal absorption of Na^+^ and eventually water by decreasing the electrochemical driving forces for apical entry of Na^+^ through epithelial sodium channels (ENaC). This occurs by inhibition of the basolateral Na^+^/K^+^-ATPase as well as K_Ca^2+^_ channels. Furthermore, exogenous H_2_S prevents the translocation of subapical vesicles containing ENaCs to the membrane thereby abrogating the action of proabsorptive, cAMP-dependent stimuli.

In contrast to secretory actions of H_2_S in the intestine, a growing body of evidence suggests that H_2_S inhibits epithelial electrolyte absorption in a variety of tissues and species [33, 62–68], including epithelia of the lung. Electrolyte absorption by pulmonary epithelial cells is facilitated by the activity of basolaterally localised Na^+^/K^+^-ATPases which establish an electrochemical driving force for the entry of sodium ions through cation channels and/or transporters in the apical epithelial membrane [69]. The concerted action of these apical entry pathways and basolateral Na^+^/K^+^-ATPases generates a vectorial absorption of sodium ions across the epithelium. Consequently, sodium absorption facilitates the paracellular absorption of chloride ions and water (Figure 4).

The rate-limiting step for sodium absorption in many vertebrate epithelia including those of the lungs is the epithelial sodium channel (ENaC) [70–73]. Experimental evidence from lung epithelial preparations suggests that exogenous H_2_S reduces ENaC-mediated epithelial electrolyte absorption (Figure 4). H_2_S-releasing compounds such as the sulfur salts NaHS and Na_2_S decreased basal ENaC-mediated sodium absorption in airway epithelial preparations from pigs and mice [62], as well as distal lung tissues from rats [33] and amphibians [63]. Consistent with the concept that lung liquid clearance largely depends on ENaC-mediated sodium transport [74–76], the sulfur salts decreased the basal rate of transepithelial liquid absorption in rat lungs [33, 68]. A decreased capacity to absorb excess lung liquid would facilitate the formation of liquid accumulation in lungs. Mice and rats develop such pulmonary oedema after exposure to exogenous H_2_S [68, 77, 78]. Furthermore, pulmonary oedema formation is characteristic for patients who suffer from acute H_2_S poisoning [79], thus providing further evidence that exogenous H_2_S reduces epithelial sodium and liquid absorption in lungs.

The precise mechanism how ENaC-mediated epithelial sodium absorption is impaired by H_2_S has not been completely clarified. Patch-Clamp and heterologous expression experiments revealed that there is no direct effect of acute H_2_S exposure on ENaC activity [62, 66, 67]. By contrast, long-term exposure to H_2_S (NaHS) reduces the expression of the ENaC *α*-subunit by ERK1/2 mediated signalling [68]. However, the observed inhibition of ENaC-mediated epithelial sodium absorption by H_2_S occurs too fast (within minutes) [33, 62, 63] to be explained by changes in gene expression of sodium transporting molecules. As mentioned above, the electrochemical driving force for apical sodium entry through ENaC is generated by the basolateral Na^+^/K^+^-ATPase. Apical membrane permeabilisation studies revealed that the activity of the Na^+^/K^+^-ATPase is reduced by H_2_S [62, 63] (Figure 4). As described, the Na^+^/K^+^-ATPase is also influenced by H_2_S in rat distal colon epithelia [45]. Furthermore, it is speculated that H_2_S-induced inhibition of sodium-uptake in larval zebrafish might be due to impaired Na^+^/K^+^-ATPase activity [65], further indicating that this enzyme is a molecular target for H_2_S.

Aside from the Na^+^/K^+^-ATPase, basolateral potassium channels contribute to the electrochemical driving force for sodium absorption by maintaining a negative membrane potential which facilitates cation influx at the apical epithelial plasma membrane. It was demonstrated that H_2_S inhibits basolateral potassium channels in lung epithelia [62, 63] (Figure 4). Inhibitor studies suggest that K_Ca_ channels are likely targeted by H_2_S; however, the precise type of basolateral potassium channel has yet to be identified. Inhibition of potassium channels as well as the Na^+^/K^+^-ATPase impairs the electrochemical gradient which is necessary for ENaC-mediated sodium influx and thereby reduces overall transepithelial sodium and, consequently, liquid absorption.

How H_2_S leads to a decreased activity of these basolateral transporting molecules remains unknown. As mentioned above, the rapid effects of H_2_S suggest changes in the activity of the transporting molecules rather than changes in their expression levels. This might be achieved by either changing their transport activity rates or, alternatively, their abundance in the basolateral plasma membrane.

In rat renal tubular epithelial cells, NaHS induces endocytosis of the Na^+^/K^+^-ATPase thereby inhibiting renal sodium absorption [64]. This occurs via a signalling cascade which is initiated by activation of the epidermal growth factor receptor by H_2_S. In H441 lung epithelial cells, there is no change in membrane abundance of the Na^+^/K^+^-ATPase within the time course of sodium transport inhibition by H_2_S [62], indicating that in lung epithelia H_2_S rather interferes with the transport activity of this enzyme.

Activity changes of transport proteins might be achieved by a posttranslational modification due to H_2_S. Initially it was suggested that H_2_S is able to directly modify thiol groups in proteins (including K_ATP_ channels), a mechanism which was referred to as S-sulfhydration [80, 81], although persulfidation is the more correct term [82]. Recently it became clear that H_2_S alone is not able to modify thiol groups, but derivatives of H_2_S such as polysulfides [83] or reaction products of H_2_S and NO such as nitroxyl (HNO) are able to do so [82]. Elegant work by Eberhardt and colleagues recently dissected a mechanism underlying the activation of a member of the TRP channel family (TRPA1) by H_2_S. These authors observed that HNO, which is formed by a redox reaction between H_2_S and NO, induced calcium influx into neurons from dorsal root ganglia of wild type, but not TRPA1-*knock-out* mice [84]. The primary targets for HNO are thiols [85] and the N-terminal region of TRPA1 contains cysteine residues which are necessary for activation of the channel by sulfhydryl-reactive agents [86, 87]. Mutation of these residues to lysine prevented the activation of human TRPA1 by HNO [84]. Furthermore, the authors demonstrated that HNO induces a formation of disulfide bonds and suggest a model by which disulfide bond formation between two cysteine pairs induces a conformational change which leads to channel opening [84].

Whether a similar mechanism would also account for the observed inhibition of the Na^+^/K^+^-ATPase and basolateral potassium channels in lung epithelia remains to be investigated. Interestingly, NO also inhibits basolateral transporting molecules in H441 lung epithelial cells, with similar kinetics to H_2_S [88], and posttranslational thiol-modification modulates the activity of the Na^+^/K^+^-ATPase [89]. Furthermore, HNO was recently shown to influence the Na^+^/K^+^-ATPase and basolateral potassium channels in distal rat colon epithelia [40], suggesting that these molecules represent molecular targets for reactive derivatives of H_2_S. However, it has to be noted that H_2_S stimulates the Na^+^/K^+^-ATPase in the rat colon, whereas an inhibition of the Na^+^/K^+^-ATPase was observed in airway surface epithelia. This discrepancy might be explained by variations in the Na^+^/K^+^-ATPase subunit composition. The Na^+^/K^+^-ATPase consists of core *α*- and *β*-subunit, of which there are four and three isoforms, respectively [90]. In addition, these core *α*/*β*-complexes assemble with an additional subunit of the FXYD protein family [91]. Tissue-dependent differences in the molecular Na^+^/K^+^-ATPase compositions might account for the different reactions to H_2_S. Furthermore, basolateral potassium channels indirectly influence Na^+^/K^+^-ATPase activity by either enhancing or diminishing basolateral potassium-recycling and hence activity of the Na^+^/K^+^-ATPase. It is possible that the potassium channel repertoire of the basolateral membrane indirectly contributes to the observed variety in responses to H_2_S.

Aside from the influence of H_2_S on basal epithelial sodium transport processes, H_2_S reduces the efficacy of proabsorptive stimuli. In lung epithelia, H_2_S abrogated the stimulation of ENaC-mediated sodium transport and lung liquid clearance by *β*-adrenergic receptor agonists [33]. This is the result of an impairment of the cAMP/protein kinase A signalling axis, although the molecular target for H_2_S remains unknown. In amphibian A6 (renal) cells, H_2_S prevented the activation of ENaC by reactive oxygen species [66] and advanced glycation end-products [67], likely due to antioxidative mechanisms.

In sum, the currently available data indicate antiabsorptive actions of exogenous H_2_S on electrolyte absorbing pulmonary epithelia.

#### 3.2.2. Endogenous H_2_S Production in Pulmonary Epithelia

In the human respiratory system, CBS/CSE have been detected in nasal mucosa [92, 93], submucosal glands [92, 93], and human H441 airway epithelial cells [33]. 3MST has only been detected at the mRNA level in human H441 cells [33]. In rodents, CBS/CSE are present in mouse airway epithelium [94], whereas CSE/3MST but not CBS were detected in rat lung homogenates [95]. All three proteins are present in airways and alveoli of cow and sea lion lungs [31]. These data indicate that pulmonary epithelia contain H_2_S-generating pathways. Consistently, using H_2_S-specific amperometric sensors it was shown that rat lung homogenates [95] as well as H441 epithelial cell lysates [33] produce detectable amounts of H_2_S.

However, H_2_S production is only detectable in the absence of oxygen (see Section 2.2.2). Consistently, inhibition of H_2_S-generating enzymes under normoxic conditions does not alter baseline sodium absorption by these cells [62]. This indicates that H_2_S is not a basal regulator of pulmonary epithelial sodium transport processes and the observed inhibition of sodium and water absorption rather represents an epithelial reaction to either exogenous H_2_S or deregulated endogenous H_2_S generation under pathophysiological conditions.

#### 3.2.3. H_2_S in Lung Epithelia: Physiological and Pathophysiological Aspects

How can these findings be integrated into a physiological context? The antiabsorptive and prosecretory epithelial responses can be interpreted as an epithelial defence mechanism against potentially harmful H_2_S concentrations which exceed the oxidative H_2_S-degrading capacities of the epithelial cells. Liquid accumulation at the luminal side of the epithelia would eliminate the potential sources of H_2_S (such as microbiota) by “flushing” of epithelia-covered compartments.

The main epithelial defence mechanism in the lung is airway mucociliary clearance [96, 97], whereas in the distal, alveolar regions of the lungs, primary defence against potential pathogens is maintained by alveolar macrophages. The entire epithelial surface of the airways is covered by a thin film of liquid, the airway surface liquid (ASL). This ASL is composed of a liquid phase surrounding the cilia of the airway epithelial cells, the periciliary liquid (PCL), and a gel-like mucus layer on top of the PCL [98]. The mucus layer is a trap for inhaled particles and potential pathogens. Due to ciliary movement, the PCL as well as the mucus layer moves towards the larynx, thereby clearing the trapped particles/pathogens from the lungs. Mucus clearance depends on the degree of hydration of the mucus gel: an increase in ASL volume enhances mucociliary clearance [96, 97, 99]. Hence, mechanisms which result in hydration of the ASL will eventually strengthen mucociliary clearance and clear the pulmonary epithelial surface from potential pathogens. The ASL volume and, consequently, the degree of mucus hydration are regulated by liquid secretion and absorption across the pulmonary epithelium.

The liquid feeding the PCL/ASL is produced by submucosal glands [96], a process which depends on vectorial anion secretion by serous cells of the glands [97]. A recent study demonstrated that submucosal glands of pig tracheal epithelia secrete liquid upon exposure to bacteria such as* Pseudomonas aeruginosa* [100], thereby showing that liquid secretion and augmented mucociliary clearance are a primary defence mechanism in the airways. Whether or not H_2_S elicits anion secretion in submucosal glands is currently unknown. In experiments addressing liquid secretion/absorption rates in fluid-filled rat lungs, there was no effect of H_2_S over that of the ENaC-inhibitor amiloride, indicating that H_2_S does not induce a detectable liquid secretion [33]. However, in this preparation the major liquid movements occur across the large surface of the alveolar regions. A potential contribution of gland secretion of the upper airways might be difficult to detect with this approach.

Aside from liquid secretion by submucosal glands, the surface epithelium of the airways is considered to absorb excess liquid [96]. This concept is supported by an anatomical view of the epithelia-covered lung structures. The alveolar surface of the human lung is by a magnitude of 10^5^ larger than that of the trachea [101]. Given that the epithelial surface liquid (alveolar liquid and ASL) is continuously transported towards the larynx, the volume of liquid lining the airway epithelia should also increase by the same magnitude. However, the height of the liquid layer lining the epithelia is relatively constant, indicating that excess volume is absorbed by the surface epithelial cells of the airways [101]. A reduction in epithelial sodium and liquid absorption would therefore result in a volume increase of the ASL. An* in vitro* approach using cultured human airway epithelia demonstrated that volume increase of the ASL results in a higher rate of mucus transport [102]. Furthermore, patients with the hereditary disease Pseudohypoaldosteronism Type 1 have a reduced ENaC activity in the airways and thus an increased ASL volume [99]. Consistently, these patients display a mucus transport rate which is profoundly above that of normal subjects [99]. This example demonstrates that a reduction in ENaC-mediated sodium absorption eventually results in an increased mucociliary clearance and would therefore be consistent with the idea of an H_2_S-induced “defence-flushing” of epithelial surfaces. The occurrence of rhinorrhoea during exposure to H_2_S [103] is consistent with an increased amount of surface liquid in the respiratory system. Furthermore, the formation of rhinorrhoea or, in the distal lung, pulmonary oedema due to H_2_S poisoning might be interpreted as a hyperresponsive epithelial defence reaction to exogenous H_2_S.

## 4. Conclusion and Perspective

The herein reviewed findings teach a concept by which physiological effects elicited by H_2_S critically depend on its concentration as well as kinetics of its production and metabolism. In accordance with Paracelsus' principle, the local concentration is the critical factor which allows the separation of physiology and toxicology of H_2_S. The precise determination of local H_2_S concentrations is still a major technical challenge (for review see [36, 37]) and hampers the classification of toxic, physiological, and beneficial roles for this molecule.

The currently available data on (1) the biochemistry of endogenous H_2_S production; (2) its correlation with oxygen availability; and (3) epithelial reactions to exogenous H_2_S suggest that normal physiology aims to maintain a low endogenous H_2_S concentration which is likely in the submicromolar range. The future challenges will be to understand pathophysiological conditions, in which these systems are impaired, and the precise determination of concentration windows which allow for pharmacological interference. Furthermore, the contribution of exogenous (i.e., microbiota-derived) H_2_S in experimental studies should be taken into consideration. At this stage it appears most suitable to close with the introductory quote:* “All things are poisons, for there is nothing without poisonous qualities. It is only the dose which makes a thing poison.”*

## Figures and Tables

**Table 1:** H_2_S-producing bacteria of the digestive tract.

Bacteria genus	Location	Process	References
*Veillonella*	Oral cavity and stomach	Metabolism of carbohydrates/lactate	[23] [24]

*Actinomyces*	Oral cavity and small intestine	Metabolism of lactate	[23] [24]

*Prevotella*	Oral cavity	Metabolism of short-chain fatty acids	[23] [24]

*Desulfosarcina, Desulfotomaculum, Desulfonema, * *Desulfovibrio (Desulfomonas), * *Desulfococcus,* * Desulfobacter *	Human and animal colon	Reduction of sulfate or other oxidized sulfur compounds using H_2_ or lactate	[25] [26] [14]
